# Supplementary material for: Peroxisome Proliferator-Activated Receptor α Attenuates Hypertensive Vascular Remodeling by Protecting Vascular Smooth Muscle Cells from Angiotensin II-Induced ROS Production
Source: Antioxidants (Basel). 2022 Nov 30;11(12):2378. doi: 10.3390/antiox11122378 (PMC9774484; doi:10.3390/antiox11122378)
Supplement: Supplementary file 1 [file antioxidants-11-02378-s001.zip › antioxidants-1984677-supplementary material.pdf]

Table S1. The composition of chow diet.

Composition	Content (%)
protein	≥18
fat	≥4
Crude fibre	≤5
Coarse ash	≤8
Water	≤10
Lysine	≥0.82
Calcium	1.0-1.8
Phosphorus	0.6-1.2
Salt	0.3-0.8

Table S2. Primer sequence.

Gene	Accession number	Primer Sequence (5'-3')
Actb	NM_007393.5	F: TTCTTTGCAGCTCCTTCGTT
		R: ATGGAGGGGAATACAGCCC
Nox4	NM_001285833.1	F: CTGGAAAACCTTCCTGCTGT
		R: TCAGGACAGATGCAGATGCT
Ppara	NM_001113418.1	F: CCCTGAACATCGAGTGCGAA
		R: TTCGCCGAAAGAAGCCCTTA

Supplementary figures

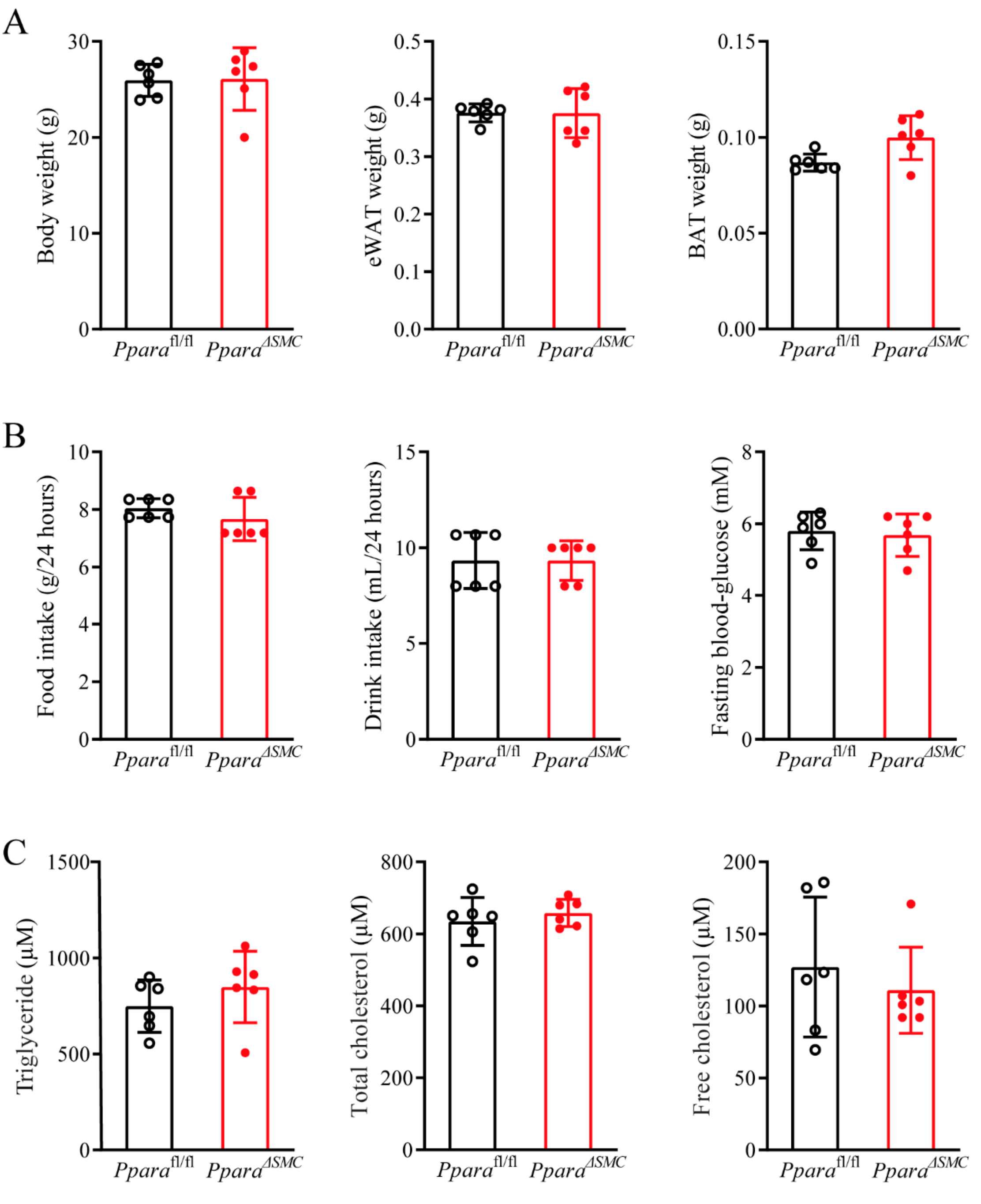

Supplementary Figure S1. The metabolic general parameters of *Ppara*$^{\Delta SMC}$ and *Ppara*$^{fl/fl}$ mice**. (A)** Body Weight, eWAT(white adipose tissue), and BAT(brown adipose tissue), **(B)** Food intake, drink intake ,and fasting blood-glucose in 2-month-old *Ppara*$^{\Delta SMC}$ and *Ppara*$^{fl/fl}$ mice**. (C)** Triglyceride, total cholesterol, and free cholesterol in plasma of 2-month-old *Ppara*$^{\Delta SMC}$ and *Ppara*$^{fl/fl}$ mice**.** (n=6).

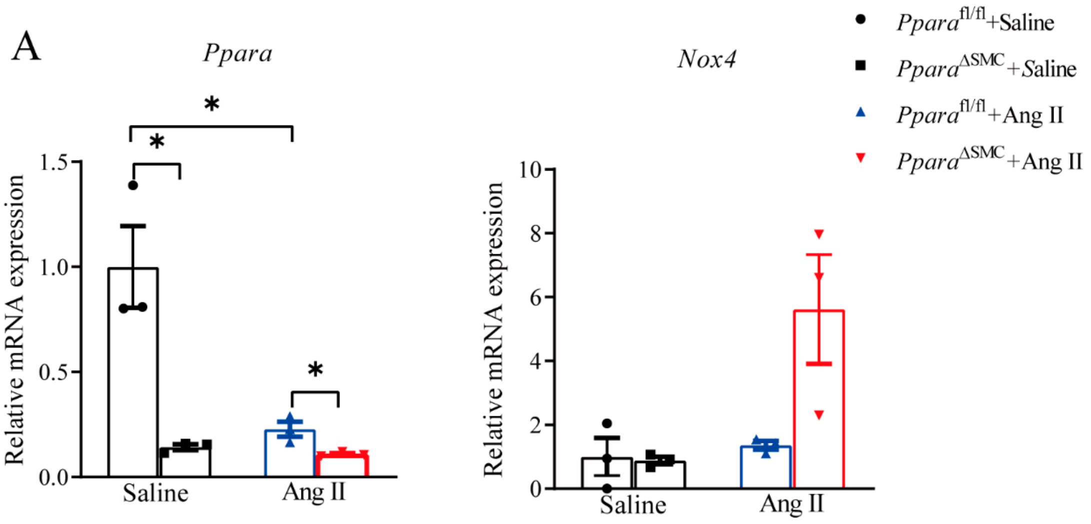

Supplementary Figure S2. PPARα deficiency in VSMCs aggravated Ang II-induced the vascular elevation of *Nox4* (A) The mRNA level of *Ppara* and *Nox4* in VSMCs from *Ppara*$^{\Delta SMC}$ and *Ppara*$^{fl/fl}$ mice were treated with Ang II (1 μmol/L) for 24 hours. (n = 3, two-way ANOVA). Data are means ± SEM. * $p < 0.05$between groups.

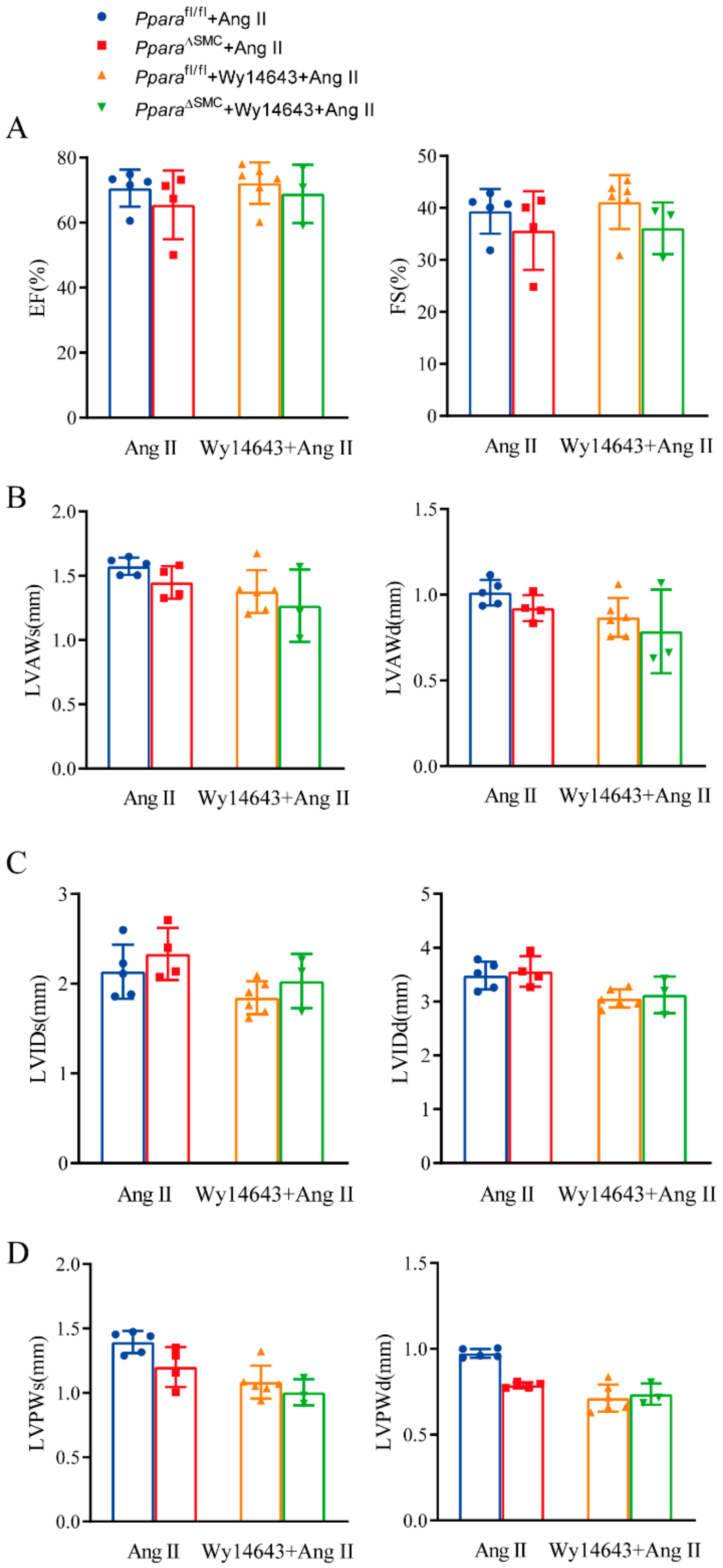

Supplementary Figure S3. Activation of PPARα by Wy14643 had no effect on Ang II-induced cardiac dysfunction. **(A)** EF and FS were assessed by two-dimensional echocardiography after Wy14643 diet. **(B)** LVAWs and LVAWd. **(C)** LVIDs and LVIDd, **(D)** LVPWs and LVPWd are recorded and analyzed (n = 3-6).
